# Interleukin-35 Regulates Immune Microenvironment of Autoimmune Hepatitis Through Inducing the Expansion of Myeloid-Derived Suppressor Cells

**DOI:** 10.3389/fimmu.2019.02577

**Published:** 2019-11-07

**Authors:** Min Lian, Jun Zhang, Li Zhao, Xiang Chen, Yanshen Peng, Qixia Wang, Shengliang Chen, Xiong Ma

**Affiliations:** ^1^Division of Gastroenterology and Hepatology, Key Laboratory of Gastroenterology and Hepatology, Ministry of Health, State Key Laboratory for Oncogenes and Related Genes, Renji Hospital, School of Medicine, Shanghai Jiao Tong University, Shanghai Institute of Digestive Disease, Shanghai, China

**Keywords:** Interleukin-35, myeloid-derived suppressor cells, autoimmune hepatitis, nitric oxide (NO), hepatic immune microenvironment

## Abstract

Interleukin-35 (IL-35) is a novel anti-inflammatory cytokine of IL12 cytokine family, however, the role of IL-35 in patients with AIH and its effect on myeloid-derived suppressor cells (MDSCs) has not yet been analyzed. The expression of IL-35 subunits (p35 and EBI3) in liver tissues was quantified by immunochemistry and its correlation with clinical parameters was explored in patients with AIH. The expression of MDSCs and IL-35 receptor (gp130 and IL-12Rβ2) were analyzed using flow cytometry and confocal staining. Besides, we utilized *in vitro* culture to explore the role of IL-35 on MDSCs expansion and activation. We found that the elevated expression of both IL-35 subunits (EBI3 and p35) in liver tissue was positively associated with degrees of hepatic inflammatory and fibrosis in patients with AIH. Furthermore, the expression of EBI3 in liver was positively correlated with patient age, serum IgG levels and serum AST, and was negatively correlated with hemoglobin and albumin. Moreover, our results showed that ratio of MDSC in peripheral blood increased significantly in AIH patients as compared with healthy controls. Further study showed that CD33, a representative marker of MDSCs, co-localized well with gp130 and IL12Rβ2, suggesting MDSCs as target cell for IL-35. Consistently, MDSCs from AIH displayed a substantial higher abundance of gp130 and IL12Rβ2 and were expanded by IL-35 *in vitro*. IL-35-induced MDSCs showed a significant increase in Nitric oxide (NO) production but not reactive oxygen species (ROS).

**Conclusions:** IL-35 might play an important role in AIH by regulating MDSCs and it could provide new insights into the therapy of AIH.

## Introduction

Autoimmune hepatitis (AIH) is an inflammatory liver disease characterized by elevated serum aminotransferase, presence of autoantibodies, hyper-gammaglobulinemia, and interface hepatitis in liver histology (1). The pathophysiology of AIH is relatively complicated and it involves complex interaction among genetics, environmental factors, and the innate and adaptive immune system (2). Although the exact pathogenesis of AIH remains unclear, emerging evidences have revealed that various cytokines play a pivotal role in the process of AIH.

Interleukin-35 (IL-35) is a newly described cytokine belonging to the interleukin-12(IL-12) family, which also includes IL-12, IL-23, and IL-27 (3–5). Similar to other IL-12 family members, IL-35 is a heterodimeric cytokine which consists of an α chain (p35) and a β chain (EBI3) (6). While the p35 subunit is ubiquitously expressed, EBI3 is selectively produced and is highly inducible (7, 8). IL-35 works through binding to its receptor (IL-35R) which consists of IL12Rβ2 and gp130, activating subsequently STAT signaling pathway (9). It has been shown that IL-35 is an important anti-inflammatory cytokine and can reduce the progression of autoimmune diseases such as collagen induced arthritis, inflammatory bowel disease, and autoimmune demyelination in central nervous system (10–12). However, the immunoregulatory roles of IL-35 in AIH have remained unexplored.

Myeloid-derived suppressor cells (MDSCs) are a heterogeneous group of myeloid progenitor cells and pathologically activated immature myeloid cells (13). Human MDSCs are phenotypically characterized as CD11b^+^CD33^+^HLA-DR^neg/low^ and can be divided into granulocytic (CD14^+^) and monocytic (CD15^+^) and lineage-negative (CD14^−^CD15^−^) subsets (14–16). Immune suppressor activity of MDSCs has been associated with arginase, inducible nitric oxide synthase (iNOS), nitric oxide (NO), and reactive oxygen species (ROS) (17). In recent years, it has become clear that MDSCs play a crucial role in the negative regulation of immune responses in chronic inflammation and autoimmune diseases. Our group previously found that MDSCs serve a beneficial role in a variety of inflammatory liver diseases by limiting T cell-mediated inflammation (18). Although a number of cytokines were shown to modulate the differentiation, expansion and activation of MDSCs, the effect of IL-35 on MDSCs remains unclear. Therefore, our study puts emphasis on investigating the expression and significance of IL-35 in AIH and the biological role of IL-35 on MDSCs.

## Materials and Methods

### Patients and Samples

A total of 120 subjects were enrolled in our study, including 36 patients with autoimmune hepatitis (AIH) (19), 27 patients with primary biliary cholangitis (PBC) (20), 18 patients with chronic hepatitis B(CHB) (21), 11 patients with non-alcoholic fatty liver diseases (NAFLD) (22), and 28 healthy controls (HC) (Table 1). In all cases, the diagnosis was established by international criteria. All individuals provided written informed consent and the protocol was approved by the Ethics Committee of Renji Hospital.

**Table 1:** Demographic and clinical profiles of study subjects.

	**AIH (*n* = 36)**	**PBC (*n* = 27)**	**CHB (*n* = 18)**	**NAFLD (*n* = 11)**	**HC (*n* = 28)**
Age (years)	55.3 (27–68)	51 (42–56)	44.5 (19–57)	34 (22–54)	44.5 (28–57)
Gender (male/female)	5/31	3/24	10/8	7/4	6/22
Hb (g/L)	124.3 (103–136)	125.5 (112–164)	137 (107–157)	135 (119–160)	126.5 (112–139)
ALB (g/L)	38.8 (24.4–44.6)	35.6 (34.5–44.5)	40.6 (34.8–47.8)	43.2 (38.8–53.2)	42 (39–45.6)
ALT (U/L)	135.5 (27–613)	55 (28–1181)	45.5 (18–164)	187 (45–249)	32.5 (23–82)
AST (U/L)	125 (30–556)	55 (26–611)	48 (18–143)	84 (26–314)	35.5 (19–50)
AKP (U/L)	149.5 (25–412)	203 (65–339)	159 (58–318)	78 (56–216)	60 (29–72)
GGT (U/L)	145.2 (21.9–343.8)	278 (17.9–1454.9)	78.8 (12.4–1378.6)	98.2 (44–310)	55 (27–88)
DBIL (μmol/L)	11.9 (2.5–157.2)	4.3 (2.5–31.3)	14.4 (2.8–32)	5.3 (4–12.9)	6.9 (4–9)
TBIL (μmol/L)	18.9 (5.4–291.2)	25.3 (5.9–56.1)	25.6 (8.6–45.5)	18.1 (11–24.1)	13 (11.4–16)
IgG (g/L)	19.6 (13.7–39.5)	12.7 (10.6–19.76)	13.8 (9.3–25.1)	10.5 (7.47–14.5)	9.6 (6.4–14.2)
IgM (g/L)	1.465 (0.88–5.49)	3.49 (0.78–7.09)	1.035 (0.8–4.47)	1.08 (0.83–1.32)	0.95 (0.68–1.2)
ANA (+/–)	36/0	3/24	2/16	1/10	NA
AMA (+/–)	0/36	27/0	0/18	0/11	NA

*All continuous variables were expressed as median (range). NA, not available*.

### Immunopathology

Paraffin-embedded liver biopsy tissues were obtained from 36 AIH patients, 27 PBC patients, 18 CHB patients, 11 NAFLD patients, and 9 Healthy Controls. Disease staging including inflammatory degree and fibrotic stages was evaluated according to the Scheuer scoring system (23). Paraffin-embedded liver tissues were stained using antibodies for IL-12p35 (short as p35 in this article), IL-12p40 (short as p40 in this article), EBI3, IL27p28 (short as p28 in this article) (Novus, Littleton, USA), gp130 (Abcam), IL12Rβ1 (Abcam), IL12Rβ2 (US Biological), IL27RA (Abcam, Cambridge, United Kingdom), and CD33 (LEICA). Briefly, after deparaffinization, specimens were incubated with 3% H_2_O_2_ for 10 min, and performed with heat induced epitope retrieval treatment by following the instructions for use of respective antibodies. After antigen retrieval, slides were incubated with 10% non-immune goat serum for 30 min and then incubated with primary antibodies overnight at 4°C. After washing with phosphate buffered saline (PBS), sections were immersed with HRP-conjugated secondary antibody (Changdao, Shanghai, China) for 30 min at room temperature, and then stained with 3, 3′-diaminobenzidine (DAB, Maixin-Bio, Guangzhou, China). Specimens were subsequently counterstained with hematoxylin for nuclear staining. Five random fields were selected in every sample and analyzed via a light microscope (Olympus, Japan). The numbers of p35, EBI3, p40, or p28 positive cells were quantified at high-power field (hpf) (40 × 10 magnification).

Confocal laser scanning microscopy was used for costaining detection of two primary antibodies between CD33 and gp130, CD33 and IL12Rβ2, or CD33 and IL12Rβ1, which were incubated together overnight at 4°C. After washing three times with PBS for 5 min, samples were incubated with Alexa 488-conjugated donkey anti-mouse IgG and Alexa 555-conjugated donkey anti-rabbit antibody (1:500) (Invitrogen/Life Technologies, UK) separately for 30 min at 37°C. The nuclei were stained by DAPI (SouthernBiotech, Birmingham, AL). Confocal scanning was performed using a laser-scanning confocal microscope (LSM-710, Carl Zeiss, Jena, Germany).

### Immunophenotype Analysis of MDSCs by Flow Cytometry

Peripheral blood mononuclear cells (PBMCs) were isolated from freshly obtained blood (AIH or HC) by Ficoll (GE Healthcare Life Sciences, Piscataway, NJ, USA) gradient centrifugation. Then PBMCs were stained with anti-HLA-DR-PercpCy5.5, anti-CD11b-PE-Cy7, and anti-CD33-BV421 (BD Pharmingen). Moreover, anti-gp130-APC (Biolegend) and anti-IL27RA-Alexa Flour488 (R&D systems) were used and then anti-IL12Rβ2 primary antibody was combined with Alexa Fluor 555-conjugated anti-rabbit IgG secondary antibody (Invitrogen/Life Technologies, UK). The percentages and numbers of HLA-DR^−/low^CD33^+^CD11b^+^ cells (MDSCs) and expression abundance of IL-35 and IL-27 receptors on MDSC were quantified and analyzed by flow cytometry (BD LSRFortessa). For some *in vitro* experiments, anti-HLA-DR-FITC, and anti-CD11b-APC (BD Pharmingen) were utilized as well. 500,000 events were recorded and analyzed using the FlowJo software version 7.2.2 (Tree Star, Ashland, OR, USA).

### *In vitro* MDSCs Culture

Human MDSCs were generated *in vitro* as described with some modifications (18). Briefly, PBMCs were cultured in T-25 flasks at 5 × 105 cells/mL in complete medium (short as CM) (RPMI 1640 medium supplemented with 10% Heat Inactivated FBS, 10 mM HEPES, 1 mM penicillin streptomycin, and 50 mM 2-mercaptoethanol) for 5 days, supplemented with cytokines as indicated, including GM-CSF (10 ng/mL, R&D), IL-6 (10 ng/mL, R&D), IL-35 (30 ng/ml, Peprotech), and IL-27 (40 ng/ml, Peprotech). PBMCs were cultured in complete medium alone as a negative control. Experiments were performed in duplicate, and culture medium and cytokines were refreshed every 2–3 days. After 5 days, all cultured cells were harvested. Adherent cells were removed by non-protease cell detachment solution Detachin (Genlantis, San Diego, CA). Cells were used for further experiments on characterization of MDSCs. For detection of NO, cells were stained with 4-amino-5-methylamino-2′, 7′-difluorofluoresce in diacetate (DAF-FM-DA) (fluorescent indicator for NO (24) and its metabolites; Invitrogen, Carlsbad, CA) and analysis was then conducted by flow cytometry. Oxidation-sensitive dye dichlorodihydrofluorescein diacetate (DCFDA) (Invitrogen, Carlsbad, CA) was used to measure ROS production (25) and the ROS content in MDSCs was analyzed by flow cytometry.

### Statistical Analysis

All continuous variables are expressed as mean ± standard error of the mean (SEM) unless specifically indicated. The Mann-Whitney *U* test was used to evaluate the differences in continuous variables. Correlations were determined by the Spearman's correlation coefficient. All analyses were two-tailed and performed using Prism software Version 6.0 (Graphpad Software, La Jolla, CA, USA). *P*-values < 0.05 were considered statistically significant.

## Results

### IL-35 Subunits (p35/EBI3) Upregulated in the Liver of AIH Patients

**Figure 1:**
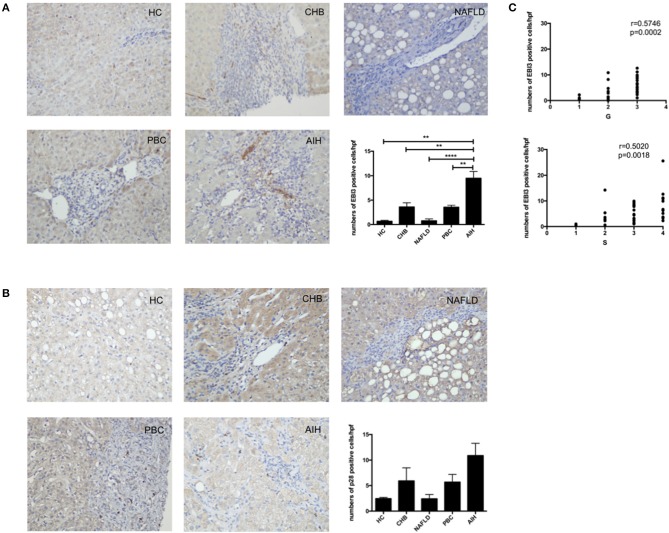
Immunohistochemistry analysis of the expression of IL-35 and IL-27 subunits in liver. Representative staining images and Statistical analysis of EBI3 **(A)**, and p28 **(B)** in HC, CHB, NAFLD, PBC, and AIH. **(C)** The numbers of hepatic EBI3+ cells were positively correlated with hepatic inflammatory degrees and fibrosis grades in AIH patients. **p < 0.01, ****p < 0.0001.

**Figure 2:**
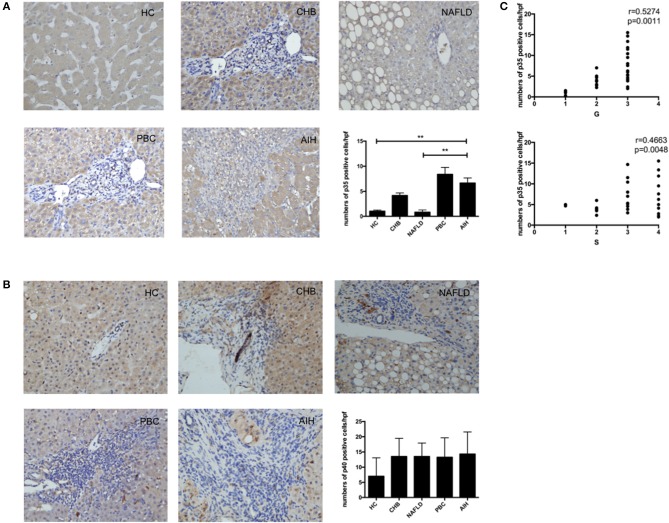
Immunohistochemistry analysis of the expression of IL-35 and IL-12 subunits in liver. Representative staining images and Statistical analysis of p35 **(A)**, and p40 **(B)** in HC, CHB, NAFLD, PBC, and AIH. **(C)** The numbers of hepatic p35+ cells were positively correlated with hepatic inflammatory degrees and fibrosis grades in AIH patients. **p < 0.01.

To confirm that IL-35 play an important role in the pathogenesis of AIH, we investigated the expression of both IL-35 subunits p35 and EBI3 in the liver by immunohistochemistry. We found that the expression of EBI3 was significantly higher in patients with AIH than that in healthy controls and patients with other liver diseases (Figure 1A). Because EBI3 also pairs with p28 to form IL-27 (26), we also investigated expression levels of p28. The expression of p28 was higher in AIH patients than that in healthy controls and other liver diseases, but this difference did not reach statistical significance (Figure 1B). Similarly, p35 expression was significantly upregulated in AIH as compared to healthy controls and NAFLD, but did not significantly differ from that in PBC and CHB (Figure 2A). Since IL-12 also share the subunit p35, the expression of another subunit of IL-12, p40 was analyzed by immunohistochemistry staining as well. No significant difference was found between above groups (Figure 2B). In addition, no correlation of the levels of p28 or p40 with the degree of hepatic inflammation and fibrosis was observed. Thus, our results indicate that IL-35, but not IL-27 or IL-12, is increased in the liver of AIH patients.

### Associations With Histopathological Evaluation and Disease Activity of Patients With AIH

Interestingly, higher level of both IL-35 subunits EBI3 and p35 was positively associated with the degree of hepatic inflammatory and the stage of hepatic fibrosis in AIH patients (Figures 1C, 2C). To further investigate the clinical significance of IL-35 in AIH, we evaluated the correlation of EBI3 and p35 expression with some laboratory parameters and patient age. The results showed that the expression of EBI3 in liver of patients with AIH was positively correlated with patient age (*R*^2^ = 0.2112; *p* < 0.01), serum IgG levels (*R*^2^ = 0.1746; *p* < 0.05) and serum AST (*R*^2^ = 0.1851; *p* < 0.05), whereas it was negatively correlated with hemoglobin (*R*^2^ = 0.3243; *p* < 0.01) and albumin (*R*^2^ = 0.1875; *p* < 0.05) (Supporting Figures 1A–E). However, there were no significant correlations between p35 expression and clinic parameters, such as age, IgG levels, AST, hemoglobin, and albumin. Therefore, our data suggested IL-35 subunit EBI3 may be a better surrogate to reflect pathological process in AIH.

### Expression of IL-35 Receptor Subunits on MDSCs

**Figure 3:**
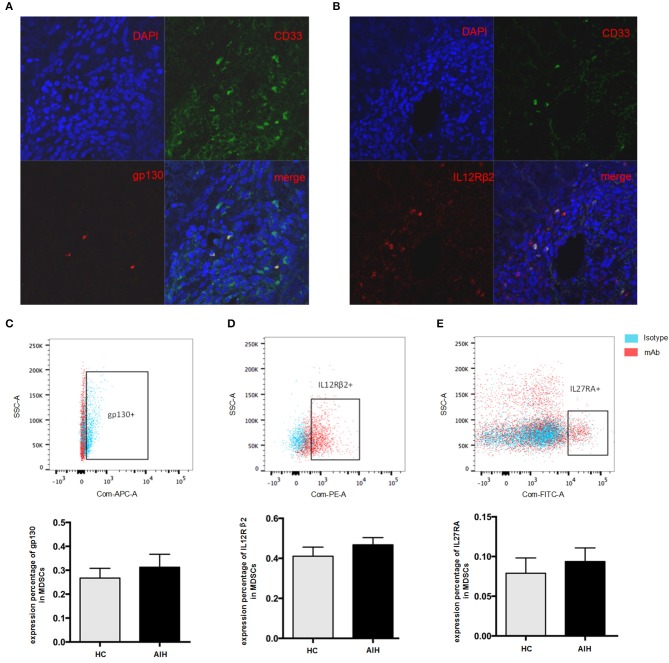
Cellular identification of IL35 receptor gp130 and IL-12Rβ2 in AIH. Confocal microscopy results showed the colocalization of MDSCs marker CD33 with gp130 **(A)** and IL-12Rβ2 **(B)** using the liver tissue of AIH patients. Representative flow cytometric results of gp130 **(C)** and IL-12Rβ2 **(D)** on MDSCs showed a substantial abundance of p35 and EBI3 receptor expression in AIH, though no significant difference was observed between AIH and controls. **(E)** Representative flow cytometric results of IL-27RA on MDSCs from HC and AIH. The average expression of p28 receptor (IL-27RA) was all <10% regardless of disease differences.

Previous data has revealed that the liver is a preferential site for MDSCs accumulation (27). Our group has previously identified MDSCs serve a beneficial role by limiting T cell-mediated inflammation in a variety of inflammatory liver diseases (18). Consistent with our previous observation (18), there were significantly increased circulating MDSCs (HLA-DR^−/low^CD33^+^CD11b^+^cells) in AIH patients as compared to healthy controls (Supporting Figure 1F). Interestingly, the immunofluorescent staining results showed that CD33, a marker of MDSCs, had a good confocal localization with gp130 and IL12 Rβ2 (Figures 3A,B), in contrast with no co-localization with IL12Rβ1(Supporting Figure 2), suggesting that MDSCs are the targeting cells for IL-35. Next, we investigated whether the IL-35 receptor (gp130 and IL12Rβ2) was detectable in human MDSCs. MDSCs from AIH displayed a substantial abundance of EBI3 receptor (gp130) expression (Figure 3C), though no significant difference was observed between AIH and healthy controls (Figure 3C). Consistently, MDSCs from AIH showed a dramatic high expression of p35 receptor (IL12Rβ2) expression (Figure 3D), while the average expression of p28 receptor (IL-27RA) was <10% in MDSCs (Figure 3E).

### Influence of IL-35 on MDSCs

**Figure 4:**
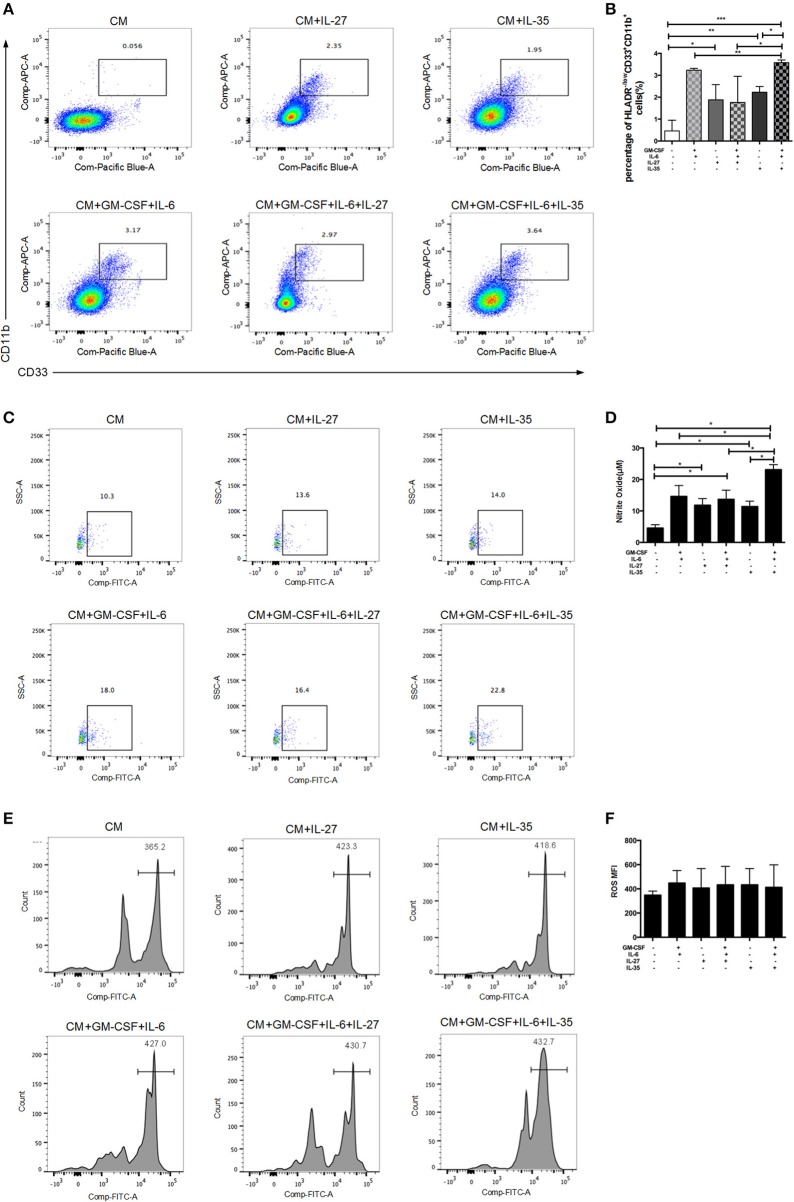
*In vitro* induction of MDSCs by recombinant IL-35 or IL-27, with or without the combination of GM-CSF and IL-6. Isolated PBMCs were cultured *in vitro* for 5 days. GM-CSF and IL-6 were added in the medium as positive controls. Classic flow cytometric charts **(A)** and statistical results **(B)** showed that IL-35 with or without GM-CSF and IL-6 effectively induced MDSCs, while IL-27 had no remarkable effects. **(C)** Representative flow cytometry charts of NO production using the DAF-DM diacetate staining. **(D)** Statistical results of flow cytometry analysis on NO produced by MDSCs. **(E)** Representative histograms of ROS production in different treatment groups. **(F)** Statistical analysis of mean fluorescence intensity (MFI) of ROS in different treatment groups. *p < 0.05, **p < 0.01, ***p < 0.001.

To test whether IL-35 can directly expand MDSCs, we stimulated human PBMCs with IL-35 or IL-27, with/without the combination of GM-CSF and IL-6. Isolated PBMCs were cultured *in vitro* for 5 days. GM-CSF and IL-6 were added in the medium as positive controls. The proportions of MDSCs were determined by flow cytometric analysis. The results showed that IL-35 with or without GM-CSF and IL-6 effectively increased the percentage of HLA-DR^−/low^CD33^+^CD11b^+^cells *in vitro*, while IL-27 had no remarkable effects (Figures 4A,B). It is well-known that NO and ROS have been implicated in MDSCs-mediated immunosuppression (17). Thus, we compared the production of these factors in MDSCs treated with and without IL-35. IL-35-expanded MDSCs showed a significant increase in NO production as compared to baseline and other cytokine treatment controls (Figures 4C,D). However, ROS production was not significantly altered upon IL-35 treatment (Figures 4E,F).

## Discussion

Since 2007, IL-35, a new immunosuppressive cytokine of IL-12 family, has been defined (4, 10). Emerging evidence has suggested that the loss of functional regulatory IL-35 can lead to enhanced immune responses, while the induction of IL-35 expression can alleviate disease symptoms of autoimmune diseases (10, 28–32). However, the expression and significance of IL-35 in AIH patients remain to be elucidated. In our current study, we have shown that the expression of EBI3 in the liver of AIH patients was significantly increased compared to those of patients with other chronic inflammatory hepatitis. Likewise, p35 expression was significantly upregulated in AIH compared to healthy controls. Moreover, patients with advanced inflammation and hepatic fibrosis grades displayed significantly elevated expression of IL-35 subunits (EBI3/p35). As EBI3 can dimerize with p28 to form IL-27, we also examined the subunit p28 of IL-27. But we did not observe any significant changes of p28 expression in AIH patients compared with healthy controls and other chronic inflammatory hepatitis. In addition, no correlation was found between levels of p28 and the degree of hepatic inflammation and fibrosis. Thus, our results indicate that IL-35, but not IL-27, is upregulated in the liver of AIH patients. Combined with these results, we speculate that the upregulation of EBI3/p35 in AIH liver tissue may be therefore attributed to induced expression of these subunits by chronic proinflammatory milieu. Previous studies found that the p35 subunit is constitutively expressed at low levels in a broad spectrum of cells (8), whereas EBI3 is selectively produced and highly inducible, and expressed in placental trophoblasts, dendritic and plasma cells, macrophages, endothelial cells, Hodgkin, and Reed-Sternberg lymphoma cells and B cell lymphomas (26, 33–37). The expression of EBI3 and p35 is increased when stimulated by the proinflammatory cytokines. Previous studies have demonstrated that IL-35 is a responsive anti-inflammatory cytokine, which is induced in response to inflammatory stimuli (38, 39). Under the inflammatory conditions, IL-35 could be upregulated to control fully-blown inflammation in the liver tissue.

To assess the potential role of IL-35 in AIH patients, we analyzed the associations of IL-35 subunits with some laboratory parameters and patient age. We found that there were positive correlations between EBI3 expression and patient age, serum IgG levels, and serum AST. In addition, negative correlations were found between EBI3 and hemoglobin and albumin. However, we could not observe significant associations of p35 with laboratory parameters and patient age. Previous reports have shown that the two subunits of IL-35 do have their own ability to regulate immunity and the process of inflammation. When they combined together to form the heterodimer, the p35 subunit may act as a ligand, and the other subunit EBI3 may mainly exert its immunological function (40). The EBI3 subunit played a potential inhibitory role in the development of autoimmune diseases (12, 32). Our results also indicate that EBI3, as part of the immunosuppressive IL-35 cytokine, plays an important role in the regulation of immune response in AIH.

Accumulating evidence shows that MDSCs are heterogeneous group of immature myeloid cells with immunoregulatory function (13). Concordant with these findings, our group has previously identified MDSCs serve a beneficial role by limiting T cell-mediated inflammation in a variety of inflammatory liver diseases (18). In our current study, we have noticed a significant increase in levels of MDSCs in AIH patients. IL-35 can act on its target cells by binging to the IL-35 receptor to transduce its signal through intracellular signaling molecule STAT-1 and STAT-4 (9). Strikingly, the immunofluorescent staining results showed that CD33, a marker of MDSCs, had a good confocal localization with gp130 and IL12 Rβ2, suggesting that MDSCs are the targeting cells for IL-35. Next, we investigated whether the IL-35 receptor (gp130 and IL12Rβ2) was detectable in human MDSCs. MDSCs from AIH displayed a substantial abundance of EBI3 receptor (gp130) and p35 receptor(IL12Rβ2) expression, though no significant difference was observed between AIH and controls. On the contrary, the average expression of p28 receptor (IL-27RA) was <10% in MDSCs. Therefore, MDSCs express receptor for IL-35, making them potentially responsive to IL-35. We thus further examined whether IL-35 can expand MDSCs. The results showed that IL-35 with or without GM-CSF and IL-6 effectively induced both the percentage and cell number of HLA-DR^−/low^CD33^+^CD11b^+^cells *in vitro*, whereas IL-27 had no remarkable effects. We observed that IL-35 rather than IL-27 could lead to the expansion of MDSCs. More importantly, IL-35-induced MDSCs were characterized by high levels of NO and low levels of ROS production. Indeed, in our previous study we demonstrated that the predominant MDSCs cell type in AIH was monocytic (CD15^+^) subset (18). G-MDSCs mediate suppression via ROS, whereas M-MDSCs mediated suppression via NO (41, 42). Therefore, MDSCs expanded by IL-35 stimulation are functional, upregulating NO expression and hence promoting the suppressive activity of inhibiting T cells proliferation in AIH. However, further studies are required to elucidate the potential molecular mechanisms.

In conclusion, we demonstrated increased expression of both IL-35 subunits in AIH liver tissue that was positively correlated with hepatic inflammatory and fibrosis. Our study also observed associations between the increased EBI3 expression and some laboratory parameters. To the best of our knowledge, our study provides the first evidence for the IL-35 receptor expression in MDSCs. IL-35 mediates the expansion of MDSCs and upregulates iNOS, which could further suppress immune responses in AIH liver. Additional studies are required to investigate the precise immunoregulatory mechanism and the signaling transduction pathway of IL-35 on MDSCs.

## Data Availability Statement

All datasets for this study are included in the article/Supplementary Material.

## Ethics Statement

This study was carried out in accordance with the recommendations of the ethics committee of Renji Hospital with written informed consent from all subjects. All subjects gave written informed consent in accordance with the Declaration of Helsinki. The protocol was approved by the same ethics committee.

## Author Contributions

ML: acquisition of data, analysis of data, and interpretation of data. JZ: samples collection and acquisition of data. LZ: analysis of data and drafting of the manuscript, study concept and design. XC, QW, and SC: performing clinical diagnosis and treatment. YP: technical support provision. XM: study concept and design, and study supervision. LZ and XM: obtained funding and critical revision of the manuscript for important intellectual content.

### Conflict of Interest

The authors declare that the research was conducted in the absence of any commercial or financial relationships that could be construed as a potential conflict of interest.

## Supplementary Material

Supporting Figure 1 Correlation analysis of hepatic IL-35 subunit EBI3 expression with clinical parameters in patients with AIH. The frequency of hepatic EBI3+ cells was positively correlated with patient age **(A)**, serum IgG levels(g/L) **(B)**, and serum AST(U/L) **(C)**, while the expression abundance of EBI3 in liver was negatively correlated with patient hemoglobin (Hb, g/L) **(D)** and Albumin (ALB, g/L) **(E)**. Moreover, the percentage of MDSCs (HLA-DR^−/low^CD33^+^CD11b^+^ cells) in PBMCs in AIH patients was significantly higher than that in healthy controls (HC)(F). ^**^*p* < 0.01.

Supporting Figure 2 Cellular identification of IL12 receptor IL-12Rβ1 in AIH. Confocal microscopy results showed the colocalization of MDSCs marker CD33 with IL-12Rβ1 using the liver tissue of AIH patients.

## Abbreviations

MDSCs: myeloid-derived suppressor cells
PBMCs: peripheral blood mononuclear cells
AIH: autoimmune hepatitis
PBC: primary biliary cholangitis
CHB: chronic hepatitis B
NAFLD: non-alcoholic fatty liver disease
HC: healthy control
AST: aspartate aminotransferase
IgG: immunoglobuin G
Hb: hemoglobin
ALB: Albumin
GM-CSF: granulocyte-macrophage colony stimulating factor
iNOS: inducible nitric oxide synthase
ROS: reactive oxygen species
MFI: mean fluorescence intensity
NO: Nitric oxide.
