# Supplementary figures and images for: Interleukin-35 Regulates Immune Microenvironment of Autoimmune Hepatitis Through Inducing the Expansion of Myeloid-Derived Suppressor Cells
Source: Front Immunol. 2019 Nov 7;10:2577. doi: 10.3389/fimmu.2019.02577 (PMC6854006; doi:10.3389/fimmu.2019.02577)

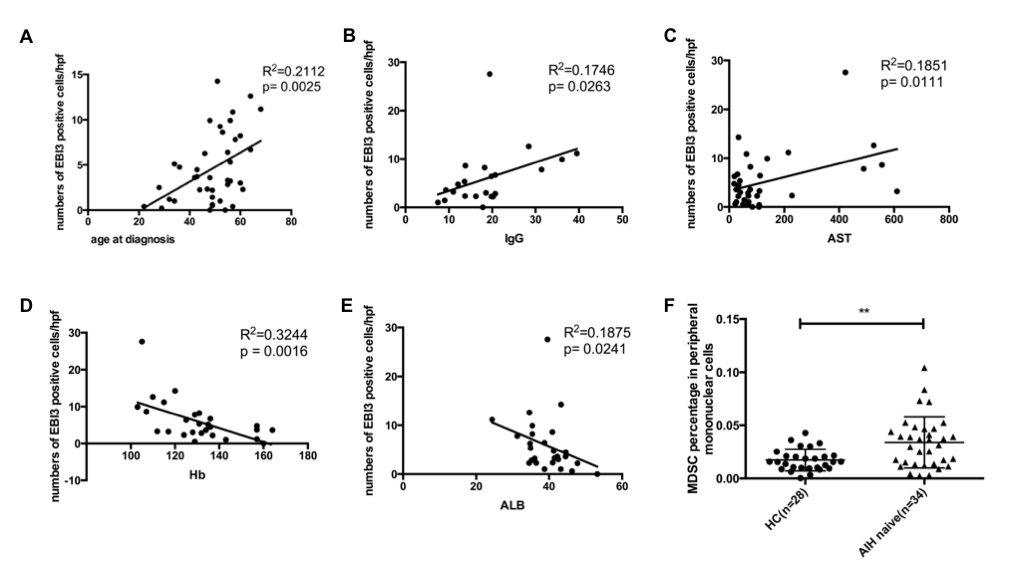

Supplement: Supporting Figure 1 — Correlation analysis of hepatic IL-35 subunit EBI3 expression with clinical parameters in patients with AIH. The frequency of hepatic EBI3+ cells was positively correlated with patient age (A), serum IgG levels(g/L) (B), and serum AST(U/L) (C), while the expression abundance of EBI3 in liver was negatively correlated with patient hemoglobin (Hb, g/L) (D) and Albumin (ALB, g/L) (E). Moreover, the percentage of MDSCs (HLA-DR−/lowCD33+CD11b+ cells) in PBMCs in AIH patients was significantly higher than that in healthy controls (HC)(F). **p < 0.01. [file Image_1.TIFF]

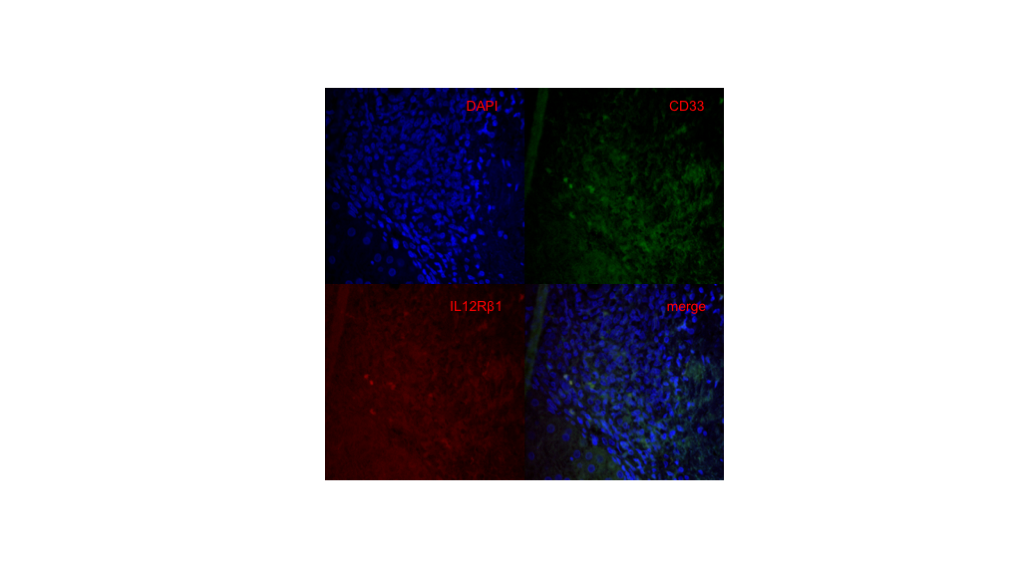

Supplement: Supporting Figure 2 — Cellular identification of IL12 receptor IL-12Rβ1 in AIH. Confocal microscopy results showed the colocalization of MDSCs marker CD33 with IL-12Rβ1 using the liver tissue of AIH patients. [file Image_2.TIFF]
